# Editorial: Practical impact of the newest achievements in assisted reproductive technologies (ART)

**DOI:** 10.3389/fmed.2022.1086972

**Published:** 2022-11-28

**Authors:** Arrigo Fruscalzo, Marie Carbonnel, Paul Pirtea, Marine Poulain, Benedetta Guani, Jean Bouquet de Joliniere, Jean-Marc Ayoubi, Anis Feki

**Affiliations:** ^1^Department of Obstetrics and Gynecology, HFR – Cantonal Hospital of Fribourg, Fribourg, Switzerland; ^2^Faculty of Medicine, University of Münster, Münster, Germany; ^3^Department of Obstetrics and Gynecology and Reproductive Medicine, Hôpital Foch, Suresnes, France

**Keywords:** assisted reproductive medicine, ovarian stimulation, infertility, hysteroscopy, fertility preservation, endometrial receptivity, uterus, fallopian tubes

In the four decades of ART history, several landmarks have stood out as turning points that have endurably changed the medical management of infertility. It is certain that these breakthroughs have recently had an impact on our daily practice of infertility management, so they deserve sets of reviews and updates that are gathered here. In this Research Topic, authors have provided a series of articles that provide a comprehensive update to the effectiveness of ARTs, their application, and concerns that might arise with their use.

## Infertility-associated complications and risk factors

The increased resort to assisted reproductive technologies (ART) has boosted the research in this field. Increasing maternal age is one of the most common features of women seeking a pregnancy through ART. Two original studies evaluated maternal and neonatal risks associated to ART and increased maternal age, whilst a third one the impact of general and life-style risk factors on fertility.

In the first study, Tai et al. compared these outcomes in spontaneous pregnancies with pregnancies achieved after ART. Authors showed that several of the most common maternal and neonatal complications, were associated to ART. These included gestational diabetes, preeclampsia, moderate or severe anemia, liver and thyroid-related pathologies, preterm birth, placenta praevia, postpartum hemorrhage, cesarean section as well as fetal growth restriction, stillbirth and fetal malformations. According to the authors, the increased risk was only partially explained by the higher incidence of multiple pregnancies in the ART group.

Bouzaglou et al. conducted a study for assessing the most common maternal and neonatal outcomes in women aged between 40 and 45 years old compared to women aged between 25 and 35 years old. As expected, later maternal age resulted to be an independent risk factor for some major obstetrical complications, including cesarean section, preterm delivery, pre-eclampsia, gestational diabetes, and intrauterine fetal demise. Authors concluded that later age patients consulting for ART should be informed of these risks.

A further manuscript focused on semen quality, as a major constraint for ART, such quality having recently and progressively degraded according to several life-style changes. Thus, developing an algorithm able to predict semen quality would be worthy of interest. Zhou et al. developed a machine learning based model toward this aim. Besides smoking status and age, several further life-style and general factors were included in this algorithm.

## Ovarian stimulation and oocytes retrieval

One of the primary adjuncts of ART, ovarian stimulation (OS), still stands today as the most effective measure ever implemented for improving ART outcomes. Yet, the equation that commands the control of the number of oocytes retrieved and embryo transferred has been drastically upended by access to embryo-vitrification.

Pirtea et al. go through and discuss the most relevant issues and advances in ART in this domain. Authors highlight in their review how the access to more flexible and secured treatments, including the use of antagonist protocols and GnRH-a for triggering oocyte maturation have reduced the risk of ovarian hyperstimulation syndrome (OHSS). On the other hand, the higher implantation rate achieved with ART has prompted physicians to switch to considering a single embryo transfer in order to reduce the incidence of multiple pregnancies.

Two further original articles of this series face two still unresolved problems of ART, the prediction of ovarian response and the prediction of developing a severe OHSS after OS.

In order to search for new parameters for predicting ovarian response to OS, Poulain et al. evaluated in their retrospective study for this scope the usefulness of calculating the follicle-to-oocyte (FOI) ratio defined by the number of retrieved oocytes divided by the antral follicular count (AFC) score (Oc/AFC). The ratio of the total number of mature oocytes (MII) and AFC (MII/AFC) was also evaluated. Results showed that an increase of all these three ratios was associated with the occurrence of live birth and implantation rate after first and cumulative embryo transfer.

Li et al. aimed in their study to search for new reliable markers of OHSS in peripheral blood samples of women undergoing a controlled ovarian hyperstimulation (COH) for oocyte retrieval. Four biomarkers of coagulation and fibrinolysis were evaluated concerning their performance in diagnostic and classification of OHSS. Results showed excellent diagnostic value, above 90%, for diagnosis of OHSS. Results were encouraging also concerning the potential to correctly classify the OHSS in mild-moderate vs. severe. According to specific cutoff values proposed, authors were able to exclude or even predict a high risk of developing severe OHSS in patients with mild–moderate OHSS. Results are noteworthy, as diagnosing and predicting the risk of developing a severe OHSS in women undergoing COH represents one of the major topics of research still in progress, with relevant medical impact in infertility treatment (Li et al.).

## Tubal factor

Tubal infertility is a further common reason for female infertility. Several non-invasive diagnostic tools have been proposed in the past in order to avert anesthesiological and surgical risks of laparoscopy, which currently remain the diagnostic gold standard.

Tan et al. evaluated in their retrospective study the predictive value of hysterosalpingograpy (HSG) compared to laparoscopy for the diagnosis of tubal factor in infertility. According to the authors HSG has a good predictive value and can be adopted as a cost-effective first-line diagnostic procedure. However, authors underline that laparoscopy should still be considered as a complementary procedure in the diagnostic work-up of infertility.

## Endometrial receptivity

The understanding of endometrial receptivity and its hormonal control has been recently updated through the study of euploid-embryo-transfers and its relationship with inflammation.

Bao et al. evaluated the potential association between ABO blood groups and immune response linked to infertility. Indeed, even though ABO blood group antigens seems to be linked to the development of several human diseases, there is still lack of consent in the current literature concerning blood group and ART outcomes. The authors found in their retrospective cohort study a positive influence of parental blood group AB compared to other blood group types on pregnancy and live birth rate in patients undergoing *in vitro* fertilization (IVF)/ intracytoplasmic sperm injection (ICSI) procedures. Considering this risk factor when counseling infertile couples seems to be noteworthy.

Chromosomal polymorphism (CPM) is considered to be a further aspect to be addressed during preimplantation genetic analysis (PGA) and ART procedures, particularly in case of unexplained recurrent pregnancy loss (uRPL). It remains, however, unclear the level at which the fertility process could be impacted. Cao et al. found that male CPM have a higher aneuploidy rate and a lower blastocyst rate compared to female CPM. Interestingly, the transfer of an euploid blastocyst with male CPM achieved the same success rate in terms of pregnancy outcomes compared to blastocysts with a female CPM. Thus, according to these results, evaluating male CPM during PGA could improve ART outcomes, at least in uRPL.

## Uterine factor

Uterine factor infertility (UFI) remains an unmet therapy—the last frontier of infertility—for which a new option, the uterine transplantation, has recently emerged. Several aspects of UFI have been reviewed, and look at cases suffering from an absence of the uterus, congenital or acquired, and/or definitive non-functionality of the uterus (Asherman Syndrome).

Favre-Inhofer et al. discuss in their review the importance of involving animal models in uterine transplantation, including the advantages and limits of each model currently in use. This overview highlights how the use of large animal models seems to be a mandatory preliminary step in order to reduce the morbidity and improve the success rate in human uterine transplantation.

Also, Sebbag et al. evaluated in their retrospective study the prevalence of intrauterine post-surgical adhesions by means of an early second-look hysteroscopy after an operative hysteroscopy. Overall, intrauterine adhesions were found in 18.7% of the women evaluated, being more frequent in women who underwent hysteroscopic lysis of adhesions (26.9%) and myomectomy (20.5), but also after polypectomy (10.9%). Data presented show a high prevalence of intrauterine adhesions and seem to support a policy of an early second-look hysteroscopy after an operative hysteroscopy.

## Fertility preservation

Finally, another major topic to be considered in ART research, fertility preservation, has been tackled. Patients at risk for premature ovarian insufficiency, both in the case of chromosomal anomalies, and in the case of toxic or immunologic impairment, need, indeed, to be properly counseled concerning current options for preserving future fertility.

Ye et al. review in their manuscript the latest knowledge concerning strategies for fertility preservation in Turner syndrome. As premature ovarian failure is a common occurrence in Turner syndrome, cryopreservation of oocytes, ovarian tissues, and eventually of embryos should be discussed as early as possible. In case the ovarian reserve is already lost, oocyte or embryo donation, gestational surrogacy, and adoption should be considered.

Chen et al. go a step further and review current knowledge on fertility preservation in case of cancer, including special situations like prepuberal status and immediate need for chemotherapy. They go through current management options in case of cancer, like ovarian tissue cryopreservation and auto-transplantation with the linked risk of also retransplanting malignant cells. Interestingly, authors also present the latest research updates in modeling an artificial ovary, combining tissue engineering and stem cell research.

## Condensation

Reproductive medicine has made constant new achievements with the development of new therapeutic options. We can undoubtedly state that today most infertility problems can be effectively treated by Assisted Reproductive Technique (ART); a fact that has remarkably reduced the number of couples who remain childless. In this Research Topic, we collected articles that provide a comprehensive update to the effectiveness of ARTs and their application to different types of infertility issues.

## Author contributions

All authors listed have made a substantial, direct, and intellectual contribution to the work and approved it for publication.

## Conflict of interest

The authors declare that the research was conducted in the absence of any commercial or financial relationships that could be construed as a potential conflict of interest.

## Publisher's note

All claims expressed in this article are solely those of the authors and do not necessarily represent those of their affiliated organizations, or those of the publisher, the editors and the reviewers. Any product that may be evaluated in this article, or claim that may be made by its manufacturer, is not guaranteed or endorsed by the publisher.

